# Mitral Annular Caseous Calcification: A Rare Variant of a Common Echocardiographic Finding Discovered with Advanced Imaging Techniques

**DOI:** 10.1155/2013/972684

**Published:** 2013-04-23

**Authors:** William L. Pomeroy, Brian Markelz, Kevin Steel, Ahmad M. Slim

**Affiliations:** Cardiology Service, San Antonio Military Medical Center, 3851 Roger Brooke Drive, San Antonio Army Medical Center, San Antonio, TX 78234-6200, USA

## Abstract

We present the case of a 90-year-old diabetic male and medically managed three-vessel coronary artery disease with evidence of an oval, nonmobile echo-density located on the posterior mitral valve annulus measuring two centimeters in diameter without significant impingement of the mitral valve on initial screening echocardiogram which was initially thought to be prominent mitral annular calcification which was later confirmed to be a rare case of caseoma as confirmed by both cardiac magnetic resonance (CMR) as well as coronary computed tomographic angiography (CCTA).

## 1. Case Report

**Figure 1:**
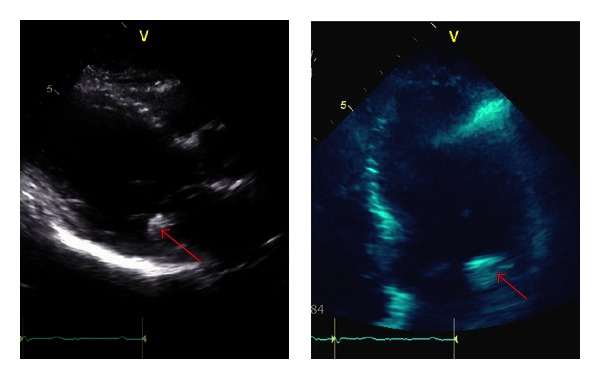
Transthoracic echo images demonstrating oval, heavily calcified echo-density on posterior mitral annulus that is difficult to differentiate from MAC (see arrow).

We present the case of a 90-year-old diabetic male and medically managed three-vessel coronary artery disease with evidence of an oval, nonmobile echo-density located on the posterior mitral valve annulus measuring two centimeters in diameter without significant impingement of the mitral valve on initial screening echocardiogram (Figure 1). 

**Figure 2:**
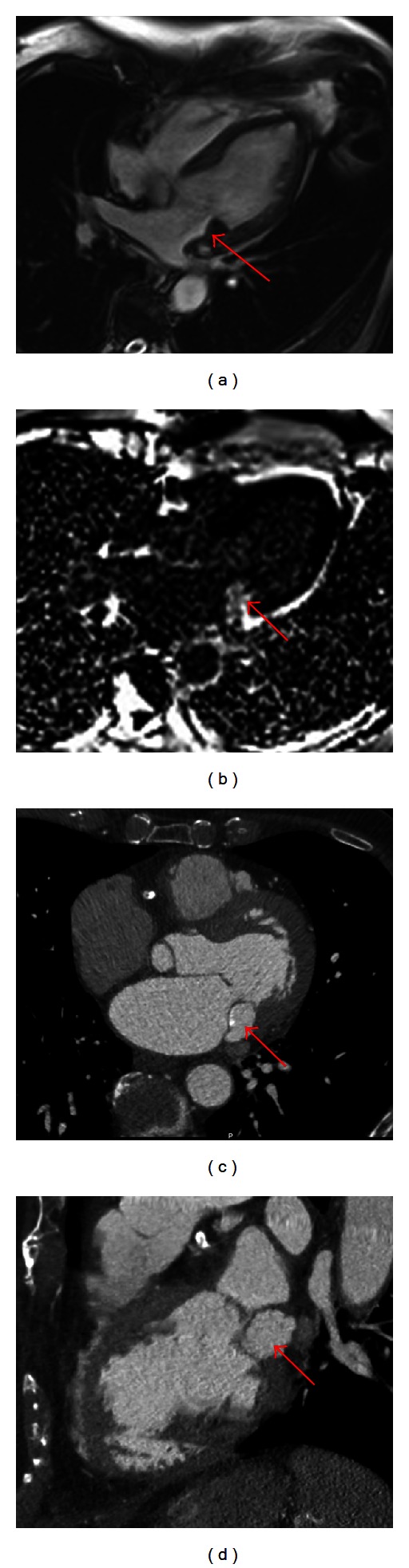
Axial CMR images demonstrating the location of the mass on the posterior annulus that usually is of low intensity signal on T2 and bright signal on T1 (a) and delayed enhancement image at the same location demonstrating external fibrosis with central liquid or proteinaceous core (b). Axial and coronal images of the caseoma with cardiac window on CCTA (*kVp = *120 with care dose variable mAs and slice thickness of 0.7mm with 0.4 overlap) with hyper-attenuated homogenous core and calcified edge with Hounsfield unit (HU) >700 ((c), (d)).

 Initially, it was thought that the mass may represent prominent mitral annular calcification, a manifestation of metastatic disease, or perhaps a primary cardiac tumor. Cardiac magnetic resonance imaging (CMR) was performed shortly thereafter which suggested the mass was either a myxoma or a metastatic tumor. Due to the patient's advanced age, comorbidities, the patient and the treating physician elected for a course of close clinical follow up with serial echocardiograms. The patient remained symptom free with minimal change to the size or echocardiographic appearance of the mass at his three and six month followup visits. Due to the possible suspicion of caseoma when repeat echocardiogram was performed and compared with CMR, a cardiac computed tomography (CCT) was performed to complement the initial imaging modalities for a confirmatory diagnosis. Multimodality imaging can be a valuable adjunct to distinguish between caseoma, tumors, or abscesses. In this instance, correlating the echocardiographic findings with imaging on CCT and CMR enabled an appropriate diagnosis of caseoma without invasive studies (Figure 2).

## 2. Discussion

The differential diagnosis of a mass located in the posterior mitral annulus includes a primary tumor, myxoma, fibroelastoma, abscess, thrombus, vegetation, or caseous necrosis of mitral annular calcification.

Mitral annular calcification (MAC) typically involves the posterior annulus and sometimes the mitral valve apparatus and is usually appreciated on transthoracic echocardiography. Recognized as part of a chronic degenerative process involving calcium deposits on the fibrous skeleton of the heart that occurs typically in patients of advanced age, MAC is a common finding on autopsy series of the heart. There is an association with MAC and cardiac conduction abnormalities as well as a higher burden of coronary artery disease and stroke. 

The presence of caseous annular mitral calcification, also known as a caseoma, is a relatively rare finding appreciated on echocardiogram. In this entity, the internal aspect of the MAC deposit becomes a liquefied mixture of calcium, cholesterol, fatty acids and forms a soft mass with a central liquid core of toothpaste-like consistency [1]. The echocardiographic prevalence is 0.6% in patients with MAC and 0.06% to 0.07% in large series of patients in all ages [2]. However, autopsy studies would suggest a higher prevalence, with up to 2.7% of patients with MAC having a caseous variant [3]. A caseoma is typically not associated with any adverse clinical effects beyond those associated with MAC.

Several misdiagnoses of caseous calcification as abscesses and cardiac tumors have been reported, in which the true nature of the variant was discovered only after surgical resection [4, 5]. Some authors speculate that the prevalence of caseous calcification may be underestimated due to the limitation of echocardiography to differentiate it from common MAC. The caseous variant of mitral annular calcification can be difficult to appreciate on transthoracic echocardiography but will typically be more oval or rounded in shape with a central area of echo lucency.

As mentioned earlier, multiple imaging modalities can be complimentary in differentiating between caseoma, tumor, or an abscess. In this instance, correlating the echocardiographic findings with imaging on CCT and CMR enabled an appropriate diagnosis without invasive studies. 

## 3. Conclusion

The differential diagnosis for a mass on the posterior mitral annulus typically includes benign or malignant tumors, metastatic disease, thrombus, or a possible infectious etiology. In the right clinical context, this differential should also include mitral annular caseous calcification, a rare variant of a common echocardiographic finding. Multimodality imaging, particularly CCT and CMR, can be very valuable in distinguishing a more benign finding from those etiologies that is more likely life threatening.
